# Urine proteome changes associated with autonomic regulation of heart rate in cosmonauts

**DOI:** 10.1186/s12918-019-0688-9

**Published:** 2019-03-05

**Authors:** Lyudmila H. Pastushkova, Vasily B. Rusanov, Anna G. Goncharova, Alexander G. Brzhozovskiy, Alexey S. Kononikhin, Anna G. Chernikova, Daria N. Kashirina, Andrey M. Nosovsky, Roman M. Baevsky, Evgeny N. Nikolaev, Irina M. Larina

**Affiliations:** 1 Institute for Biomedical Problems – Russian Federation State Scientific Research Center Russian Academy of Sciences, Moscow, Russia; 2 V.L. Talrose Institute for Energy Problems of Chemical Physics, Russian Academy of Sciences, Moscow, Russia; 3 Moscow Institute of Physics and Technology, Dolgoprudny, Moscow region Russia; 4 Skolkovo Institute of Science and Technology, Skolkovo, Moscow region Russia

## Abstract

**Background:**

The strategy of adaptation of the human body in microgravity is largely associated with the plasticity of cardiovascular system regulatory mechanisms. During long-term space flights the changes in the stroke volume of the heart are observed, the heart rate decreases, the phase structure of cardiac cycle is readjusted The purpose of this work was to clarify urine proteome changes associated with the initial condition of the heart rate autonomic regulation mechanisms in cosmonauts who have participated in long space missions. Urine proteome of each cosmonaut was analyzed before and after space flight, depending on the initial parameters characterizing the regulatory mechanisms of the cardiovascular system.

**Results:**

The proteins cadherin-13, mucin-1, alpha-1 of collagen subunit type VI (COL6A1), hemisentin-1, semenogelin-2, SH3 domain-binding protein, transthyretin and serine proteases inhibitors realize a homeostatic role in individuals with different initial type of the cardiovascular system regulation. The role of significantly changed urine proteins in the cardiovascular homeostasis maintenance is associated with complex processes of atherogenesis, neoangiogenesis, activation of calcium channels, changes in cell adhesion and transmembrane properties, changes in extracellular matrix, participation in protection from oxidative stress and leveling the effects of hypoxia. Therefore, the concentrations of these proteins significantly differ between groups with dominant parasympathetic and sympathetic influences.

**Conclusion:**

The space flight induced urine proteome changes are significantly different in the groups identified by heart rate autonomic regulation peculiarities before space flight. All these proteins regulate the associated biological processes which affect the stiffness of the vascular wall, blood pressure level, the severity of atherosclerotic changes, the rate and degree of age-related involution of elastin and fibulin, age-related increase in collagen stiffness, genetically determined features of elastin fibers. The increased vascular rigidity (including the aorta) and of myocardium may be regarded as a universal response to various extreme factors. Significant differences in the semi-quantitative analysis of signal proteins between groups with different types of autonomic regulation are explained by a common goal: to ensure optimal adaptation regardless of age and of the genetically determined type of responses to the extreme environmental factors effects.

**Electronic supplementary material:**

The online version of this article (10.1186/s12918-019-0688-9) contains supplementary material, which is available to authorized users.

## Background

The strategy of adaptation of the human body in microgravity is largely associated with the plasticity of cardiovascular system regulatory mechanisms. The leading factor in the appearance of hemodynamic changes in the cosmonaut’s body is microgravity. The main hemodynamic changes are associated with redistribution of blood in the thoraco-cranial direction [1, 2]. A new hemodynamic situation leads to changes in the mechanisms of autonomic control of circulation, and to the restructuring of the relationship between the central and peripheral blood circulation. During long-term space flights (SF) the changes in the stroke volume of the heart are observed, the heart rate (HR) decreases [3], the phase structure of cardiac cycle is readjusted [4].

Overdistension of cardiomyocytes and changes in vascular wall stiffness and elasticity lead to changes in signal proteins. The detected in the blood and urine proteome intra - and extracellular regulators of endothelial function, of cell adhesion, of extracellular matrix condition and of lipogenesis activation play a great role in this process [5]. Protein signaling molecules, such as C-reactive protein, homocysteine, fibrinogen, apolipoproteins, sST2 and others are the markers of adaptation stages (initial, in development, or decompensation). Interleukin 1, 3, 5, 8, tumor necrosis factor, insulin-like growth factor become very important. These features of the proteome correlate not only with structural and hemodynamic characteristics (ejection fraction, volume and structural parameters of myocard), but can be the markers for identification of patients at risk for acute vascular and neurological pathology.

The purpose of this work was to clarify urine proteome changes associated with the initial condition of the heart rate autonomic regulation mechanisms in cosmonauts who have participated in long space missions. Urine proteome of each cosmonaut was analyzed before and after space flight, depending on the initial parameters characterizing the regulatory mechanisms of the cardiovascular system.

## Materials and methods

The objects of the study were urine samples and 5-min samples of electrocardiogram (ECG) at rest in twelve male cosmonauts (age 46.5 ± 3.4 years), who performed SF with duration 169–199 days on board the Russian segment of the International space station (ISS) and who had the same dates of pre-flight ECG studies and collection of urine samples. The health of cosmonauts was assessed by the medical expert Commission of Institute for Biomedical Problems Russian Academy of Sciences (IBMP RAS), according to international criteria.

All members of the main and backup crews provided written informed consent to participate in the experiment “Proteome” in advance of their missions on the ISS. The experiment “Proteome” was approved by Biomedicine Ethics Committee of IBMP RAS/Physiology Section of the Russian Bioethics Committee Russian Federation National Commission for UNESCO and Human Research Multilateral Review Board, NASA, Houston, Tx, USA.

Collection of urine samples was carried out in the framework of the experiment “Proteome” on 30–45 days before start and on 1st and 7th day after landing. Urine collection was carried out at day time after the usual for the tested person breakfast, in the form of a freely detachable 2nd fraction, which was subsequently prepared for mass spectrometric analysis, according to the standard protocol [6]. Urine samples were subjected to preparation, consisting of stages: recovery, alkylation, protein deposition and proteolysis using trypsin.

The shotgun proteomics methodology was used. The tryptic peptides mixture was separated by liquid chromatography (Agilent 1100, Agilent Technologies Inc., Santa Clara, USA) and analyzed by 7 T LTQ-FT Ultra hybrid mass spectrometer (Thermo, Bremen, Germany). A column with reversed phase ReproSil-Pur C18 (particle diameter 3 μm, pore diameter 100 Å, Dr. Maisch GmbH, Ammerbuch-Entringen, Germany) has been used for chromatography, manufactured using a capillary emitter (Pico-tip, New Objective Inc., USA). The data-dependent mass spectrometric analysis was performed using the Xcalibur software (Thermo Electron, Bremen, Germany) in a two-stage mode.

Proteins were identified with the help of MaxQuant software, using the SwissProt_2016_06 database. Only proteins identified by more than two peptides and with at least one unique peptide were considered as identified. Label free analysis for protein quantitation was performed using MaxQuant software. This included quantification of peptides recognized on the basis of mass and retention time but identified in other LC MS/MS runs (method “without label” with additional option “match between the runs” was used for semi-quantitative analysis). MaxQuant computes protein intensities as the sum of all identified peptide intensities (maximum detector peak intensities of the peptide elution profile, including all peaks in the isotope cluster). Protein intensities were divided by the number of theoretically observable peptides (calculated by in silico protein digestion with a PERL script, all fully tryptic peptides between 6 and 30 amino acids were counted while missed cleavages were neglected). All LC-MS/MS raw files and MaxQuant data are uploaded in proteome data repository (PRIDE) and an internal ID of:[px-submission #305348] has been assigned.

ECG registration was conducted in the framework of “Pneumocard” experiment at 30–45 days before start. The cardiovascular regulatory mechanisms condition was assessed in 5-min ECG samples at rest in supine position. The time and frequency domain analysis of heart rate variability (HRV) was carried out according to the recommendations developed by the European cardiological and North American electrophysiological Societies (1996).

The principal component method was used for statistical analysis. The statistical hypothesis that the examined sample was taken from the normal distribution was tested. The statistical Shapiro-Wilkes test was used for this purpose. This test is one of the most effective tests of normality, and *p* value was < 0.325, i.e., the null hypothesis of belonging to the normal distribution was not rejected. The differences between the experimental samples were found using the Tukey’s honestly significant difference test.

The Perseus software package was used to determine molecular functions and biological processes involving the identified proteins. For determination of protein-protein interactions STRING database was used. The interactions include physical and functional associations; they stem from computational prediction, from knowledge transfer between organisms, and from interactions aggregated from other databases. Detected proteins were annotated using GeneOntology Database.

## Results and discussion

### Heart rate variability analysis in background studies before space flight

Heart rate variability is the most accessible indicator of the whole body condition and of the regulatory controlling mechanisms influence on the cardiovascular system in particular. Therefore, we have made an attempt to find a correlation between characteristics of the heart rate regulation and the proteome signal proteins involved in the major processes of adaptation. Depending on the heart rate (HR) and the autonomic regulation parameters in background studies, the subjects were classified into two groups (each consists of 6 subjects): HR in the first group was 60,12 ± 2.21 bpm (from 52,9 to 68,3 in different persons), in the second group – 75,02 ± 3.31 bpm (from 65,3 to 88,8 in different persons).

The results obtained in background studies suggest the dominance of different types of autonomic regulation in cosmonauts from the classified groups. The difference of the pNN50 (a measure of the parasympathetic activity) and SI (an indicator of regulatory systems stress, reflecting the central mechanisms prevalence over the autonomous) indices illustrates this fact. Spectral characteristics of the heart rate variability (HRV) - the low-frequency and high-frequency HRV components ratio (LF/HF) which reflects the relative activity of the subcortical sympathetic nervous center, and the values of the HRV spectrum total power (TP) confirm the above statement (Table 1, Additional file 1).Thus, parasympathetic effects on the heart rate have prevailed in the first group during background studies, and sympathetic effects - in the second, which allowed to search for signal proteome proteins, also significantly different in these groups in the background and on + 1 and + 7 days of the period of readaptation to terrestrial conditions. This was the starting point for further data analysis.

**Table 1:** HRV parameters in background studies before space flights

Parameters	HR	pNN50	SI	LF/HF	IC	TP
Group 1	60,12 ± 2,21	13,73 ± 4,30	60,89 ± 12,58	2,47 ± 0,62	4,23 ± 1,08	2609,76 ± 573,07
Group 2	75,02 ± 3,31	1,01 ± 0,84	227,66 ± 46,18	4,00 ± 1,29	6,28 ± 1,96	699,03 ± 169,26

Totally around 200 core proteins were determined in urine samples, 34 of which were statistically significantly changed in the entire sample of 12 cosmonauts (*p* < 0,01) at the first day after space flight when compared with background data. In addition, 28 proteins changed when comparing + 1 and + 7 days of the recovery period and 14 – when comparing the background and + 7 day, respectively (Additional files 1 and 2).

In accordance with the classification into two groups by the pre-flight peculiarities of autonomic regulation of heart rate 8 proteins were revealed from the proteins list which are significantly different in groups (*p* < 0,05) at different points of the study (Statistica 10 software).

### Urine proteome changes before space flight in individuals with different type of autonomic regulation

**Fig. 1:**
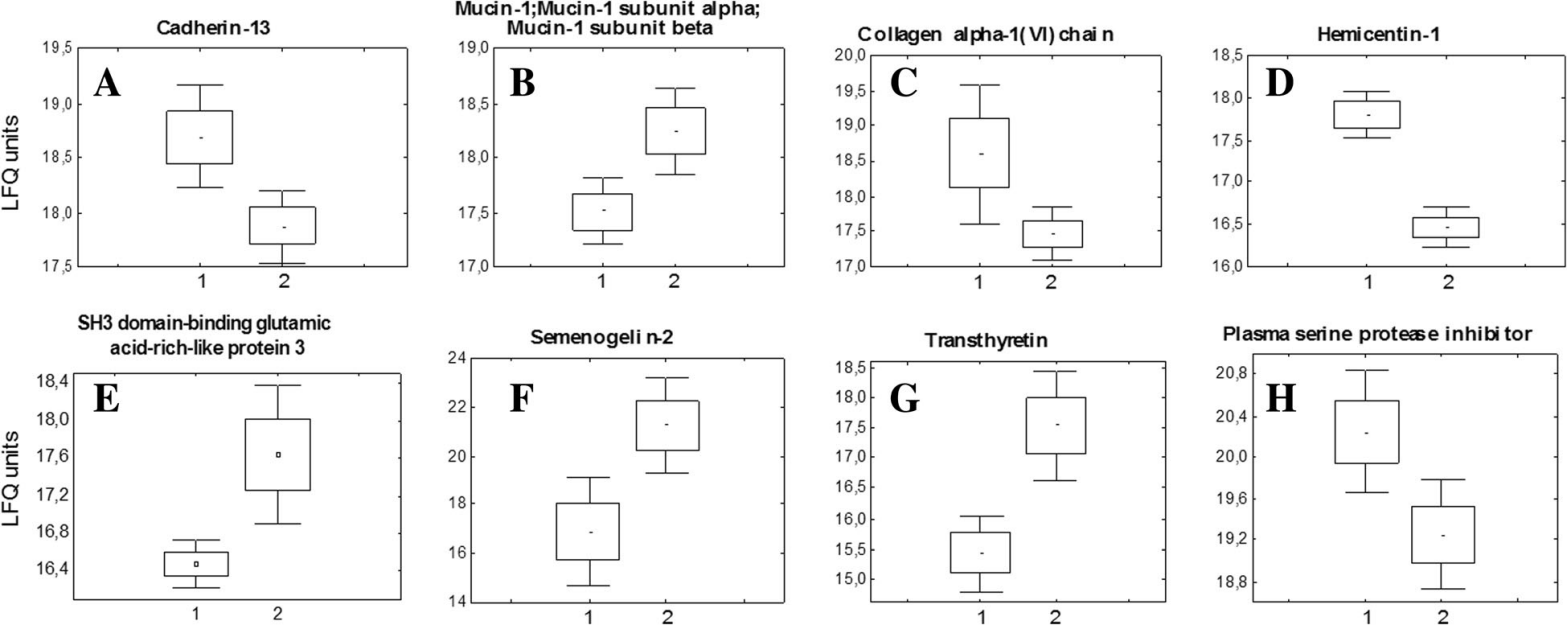
Significantly changed urine proteins in two groups with different type of autonomic regulation. Before space flight: **a** T-cadherin; **b** mucin-1; **c** alpha-1 subunit of collagen type VI (COL6A1); at + 1 day after space flight: **d** hemicentin-1, **e** SH3 domain-binding glutamic acid-rich-like protein 3, **f** semenogelin-2; at + 7 day after space flight: **g** transthyretin, **h** plasma serine protease inhibitor. Label-free quantitation (LFQ) values are plotted along the vertical axis for each protein in two groups (1, 2)

In the background period, proteins cadherin-13, mucin-1, alpha-1 of collagen subunit type VI (COL6A1) significantly differ between groups with different type of autonomic regulation. Intergroup differences between hemisentin-1, semenogelin-2, SH3 domain-binding protein, transthyretin and serine proteases inhibitors are reliable also at all points of the study (*p* < 0,05), see Fig. 1a-h.

Cadherin-13 (CDH13) belongs to the cadherin superfamily, a group of encoding calcium-dependent molecules adhesion genes, with a wide range of biological effects as interstitial regulators of signaling pathways. Experimental studies have shown that changes in T-cadherin expression in vascular cells are associated with restenosis and atherosclerosis [7, 8], that is, with the involvement of T-cadherin in atherogenesis, angiogenesis, and changes in vascular wall stiffness and elasticity [9]. In Rubin’s and co-authors studies, on models in vitro and in vivo, it was shown that T-cadherin is a “repellent molecule” and inhibits angiogenesis at the level of endothelial cell migration and the formation of small vessels and capillaries. These effects are based on the homophilic interaction between T-cadherin molecules [10]. It was found that T-cadherin is a LDL receptor mediating the activation of intracellular signaling involving [Ca2+] and cell migration along the low density lipoprotein gradient [11]. Maximum T-cadherin expression in CDH13 protects vascular endothelial cells from apoptosis under oxidative stress [12]. T-cadherin is a factor influencing endothelial barrier function by regulation of endothelial permeability. T-cadherin is a navigational molecule that mediates the negative regulation of blood vessel growth. T-cadherin suppresses the initial stages of angiogenesis, but does not affect the processes of blood vessels maturation. Its expression increases in such pathological conditions of vessels, where the endothelial dysfunction develops [13]. The T-cadherin receptor of endothelial and vascular smooth muscle cells is involved in the development of hypertension [14]. Associations of cardiovascular and metabolic diseases with T-Cad gene polymorphism, predominant T-Cad expression in the cardiovascular system, cardioprotection and revascularization of ischemic limbs in the interaction of T-Cad with adiponectin indicate the role of this receptor in the functioning of heart cells and vessels most important [15].

Mucin 1 (MUC1) is a membrane - bound glycoprotein whose functions include cell adhesion modulation, signal transduction, surface-active properties, hydration of epithelial surfaces and their protection from infection [16]. Hanson and Hollingsworth show that the main function of the morphogenetic signaling of MUC1 is to help reprogram the gene expression in response to changes in cell morphology (for example, the loss of cell polarity observed with changes in gravity and magnetic environment). The effect of MUC1 is regulated by cytokines or interstitial growth factors that appear in tissue damage, inflammation, or remodeling [17]. MUC1 enhances the formation, proliferation and migration of endothelial cells in hypoxia. Moreover, hypoxia-induced MUC1 partially regulated two other induced by hypoxia proangiogenic factors, including vascular endothelial growth factor A and thrombocyte growth factor [18]. Our findings about this signal protein, with regard to the effect of SF factors combination correspond the previously described dynamics of this protein in the study of bedrest influence on the endothelium [19].

The alpha-1 subunit of collagen type VI (COL6A1) - a component of connective tissue and extracellular matrix (ECM). ECM, as a stroma component, does not perform only the function of supporting the cells, but also plays a dynamic role in metabolic processes that affect cell proliferation, differentiation, apoptosis, as well as deposites biological active growth factors [20]. The myocardial ECM has a complex organization, its own system of regulation and reproduction, and is able to respond quickly to changes in the load on the heart. It was ascertained that the change of ECM structure is observed in angiogenesis, tissue involution, regeneration, etc. ECM plays a significant role in the progression of heart failure [21]. Zhuang and colleagues identified several potential biomarkers for dilated cardiomyopathy, including alpha-1 subunit of collagen type VI [22]. Cardiac remodeling is a pathological process that develops after any pathological process and changes the shape, size and function of the heart cavities. Functional and structural changes of the myocardium are widely associated with changes in the course of inflammation and the condition of the extracellular matrix [23].

Collagen plays an important role in maintaining the integrity of tissues. Several studies have shown that collagen VI also suppresses the deposition of amyloid β-peptides. Collagen VI has a protective effect, affecting apoptosis and autophagy, two key intracellular processes of primary importance for central nervous system homeostasis and the neurodegenerative diseases genesis [24]. The lack of collagen VI causes impaired muscle regeneration and reduces the self-renewal possibility of satellite cells after injury [25]. Smeriglio and colleagues have shown that collagen VI is important for cartilage regeneration in osteoarthritis [26]. It was found that the dominant Col 6 gene mutations are a common cause of congenital Ulrich muscular dystrophy [27]. Interestingly, that this protein is observed in the study of age-dependent changes in the proteome. Therefore, the presence of collagen VI can be not only a marker of connective tissue adaptation, but also a signal of the “age” factor.

### The urine proteome changes at + 1 day after space flight in individuals with different type of autonomic regulation

On the + 1 day after flight two groups significantly differ in: Hemicentin-1 (Fig. 1d), SH3 domain-binding glutamic acid-rich-like protein 3 (Fig. 1e), semenogelin II (Fig. 1f) and the alpha-1 subunit of collagen type VI (COL6A1) is detected again, which indicates adaptive processes at the cellular and tissue level due to landing.

Hemicentin-1 (HMCN1) belongs to extracellular matrix proteins, many of which present in large amounts in the cardiovascular system [28]. This proteins support the architectonics of adhesive and flexible compounds of epithelial cells, participate in myocardial remodeling, and influence the migration of cardiac fibroblasts. Fibulin-6 as integral and essential component of the ECM contributes not only to ECM homeostasis, but also regulates the activity of fibroblasts by regulating TGF-β [29]. Myocardial remodeling and fibrosis which lead to heart failure are characterized by a pronounced TGF-β induction and activation. This is the “main switch” in the myocardial infarction and heart failure pathogenesis, as well as in hypertrophic and ischemic human cardiomyopathy. Many proteins, such as fibrillin 8, fibronectin 9 and decorin 10, are the key for TGF activity. The previously described age-dependent dynamics of this protein is inextricably associate with cardiosclerosis, fibrosis and other structural rearrangement ofmyocard, which corresponds with the opinion of other authors. Hemicentin-1 is also associated with age-related macular degeneration, the main cause of vision loss in the elderly [30, 31]. So, hemiсentin-1 is an important component of the extracellular matrix, which determines the structure of cellular contacts in tissues exposed to deformation, which is especially important when the launch, space flight and landing factors affect the cardiovascular system condition and the vascular components of nervous system.

The SH3 domain-binding glutamic acid-rich-like protein 3 (SH3BGRL3) is an RNA-binding protein that coordinates signal transduction and post-transcription regulation of genes. The G3BP has been shown to be involved in cellular processes such as cell survival, growth, proliferation, and apoptosis. It is very important that this protein is activated in cardiomyocyte hypertrophy, thus, the SH3BP1 detection is a marker of the normal adaptation processes failure and of transition to the stage of compensatory hypertrophy [32]. The SH3BP1 may also act as a modulator of the glutaredoxin biological activity mainly in the smooth muscles and immune cells, it protects cells from induced by TNF apoptotic lysis [33]. This protein is a known regulator of arteriosclerosis [34]. The analysis of biological effects makes it evident the association of the SH3BP1 activity and alkaline phosphatase, chromostatin, osteopontin, which indicates about its effects on cartilage and bone tissue metabolism.

Semenogelin II (SEMG2) is involved in the zinc ions transport, antibacterial protection, activation of hyaluronidase and regulation of sperm movement. It was revealed that the semenogelins overexpression led to a significant increase in the membrane mitochondrial potential, which indicates an increase in the cells energy status. The potential biological role of semenogelins in the regulation of oncoassociated metabolism and oxidative stress has been shown [35]. The detection of semenogelin II in the men’s urine proteome may be of a dual nature. On the one hand - it is possible a presence of seminal fluid traces in the samples (normal variant), on the other hand, a protein semenogelin II was found among the structural proteins of renal tissue in the urine of healthy people in 86.7% of cases. It is known that the decrease in the semenogelin II expression intensity in urine of patients with chronic heart failure in the presence of renal damage indicated the reduction in the nephrocytes matrix structural gel formation [36]. The obtained data require further analysis and clarification of the results interpretations.

### The urine proteome changes at + 7 day after space flight in individuals with different type of autonomic regulation

A significant difference between groups of two proteins - transthyretin (Fig. 1g) and of plasma serine protease inhibitor (Fig. 1h) remains at + 7 days period of readaptation. It is important and we propose that this fact indicates the activity of adaptation biological processes with the participation of these signal proteins within seven days of the readaptation period.

Transthyretin (TTR) - is a serum protein with a tetrameric structure, it is produced in the liver, retina and cerebral vascular sheath predominantly. TTR not only carries out the transport of vitamin and thyroid hormones but also participates in their transfer through the blood-brain barrier. Transthyretin, as a key protein of cerebrospinal fluid, is considered as a marker of the blood-brain barrier dysfunction. This prealbumin is a transport protein of thyroxine. It is not known whether its level increases when exposed to radiation, as a result of the synthesis activation in the liver, or the acceleration of cerebrospinal fluid metabolism, but this leads to the activation of the thyroxine deposition and reducing the availability of its free form for target cells. On the other hand, transthyretin contributes to the precipitation of amyloid-like fibrils in different tissues, depositing them in the light chains of immunoglobulins, including in the myocardium. Thus, a certain type of the extracellular matrix structure violation is formed. Homeostasis and extracellular matrix composition are determined, in particular, by specific ways of collagen destruction, which are controlled by the matrix metalloproteinases balance and their inhibitors in tissues. The data, obtained by histological examination of tissues from dogs exposed to chronic irradiation at a dose of 21 to 188 cGy (mean tissue dose per year) for 3–6, indirectly indicate the possibility of extracellular matrix dysfunction involvement in radiation impact.

The dynamics of this protein during space flight can be explained by changes in blood filling of parenchymal and gliotic structures of the liver, retina and brain under the influence of the main factors of microgravity and overloads during launch– flight – landing stages.

It is interesting, that the transthyretin molecules of “wild type” and the mutant forms of this protein, can, under certain conditions (including radiation and hypomagnetic environment in SF), change the spatial configuration, contributing the appearance of conformational changes. The progression of conformational changes in the transthyretin molecules is accompanied by their aggregation and formation of ordered filamentous insoluble structures — amyloid fibrils, which lead to the development of systemic amyloidosis when accumulate in the intercellular space of organs and tissues. Transthyretin (TTR) cardiac amyloidosis is an unappreciated factor for heart failure in elderly patients. While the diagnosis usually requires to make tissue biopsy and to demonstrate the amyloid histologically, cardiac amyloidosis can also be identified noninvasively by echocardiography and cardiac magnetic resonance imaging (MRI). The clinical treatment TTR cardiac amyloidosis differs from other forms of heart failure, so, the disease recognition is important [37].

The plasma serine protease inhibitor (IPSP) or endothelial protein receptor C (EPCR) - is expressed on the endothelial cells of the heart and lungs arteries, as well as of the lungs and skin. The EPCR enhances the protein C activation under the action of the thrombin-thrombomodulin complex participating in the transmission of a mediated by protein C signal, controlling blood clotting. The activated protein C has an anti-inflammatory effect on endothelial cells and macrophages by inhibiting the release of proinflammatory mediators and reducing the vascular endothelial adhesion molecules expression. This protein protects cells from apoptosis and stabilizes the endothelial barrier, including renal tissue podocytes. The plasma serine protease inhibitor (IPSP) is increased at + 1 day in the blood proteome and continues to grow at + 7 days in the blood and urine proteome in the recovery period. It is known that IPSP is involved in the intravascular and extravascular proteolytic activity regulation. We hypothesized that the synthesis of protease inhibitors such as IPSP is activated after spaceflight to deactivate the proteolysis which is caused by a combination of SF and landing factors.

**Fig. 2:**
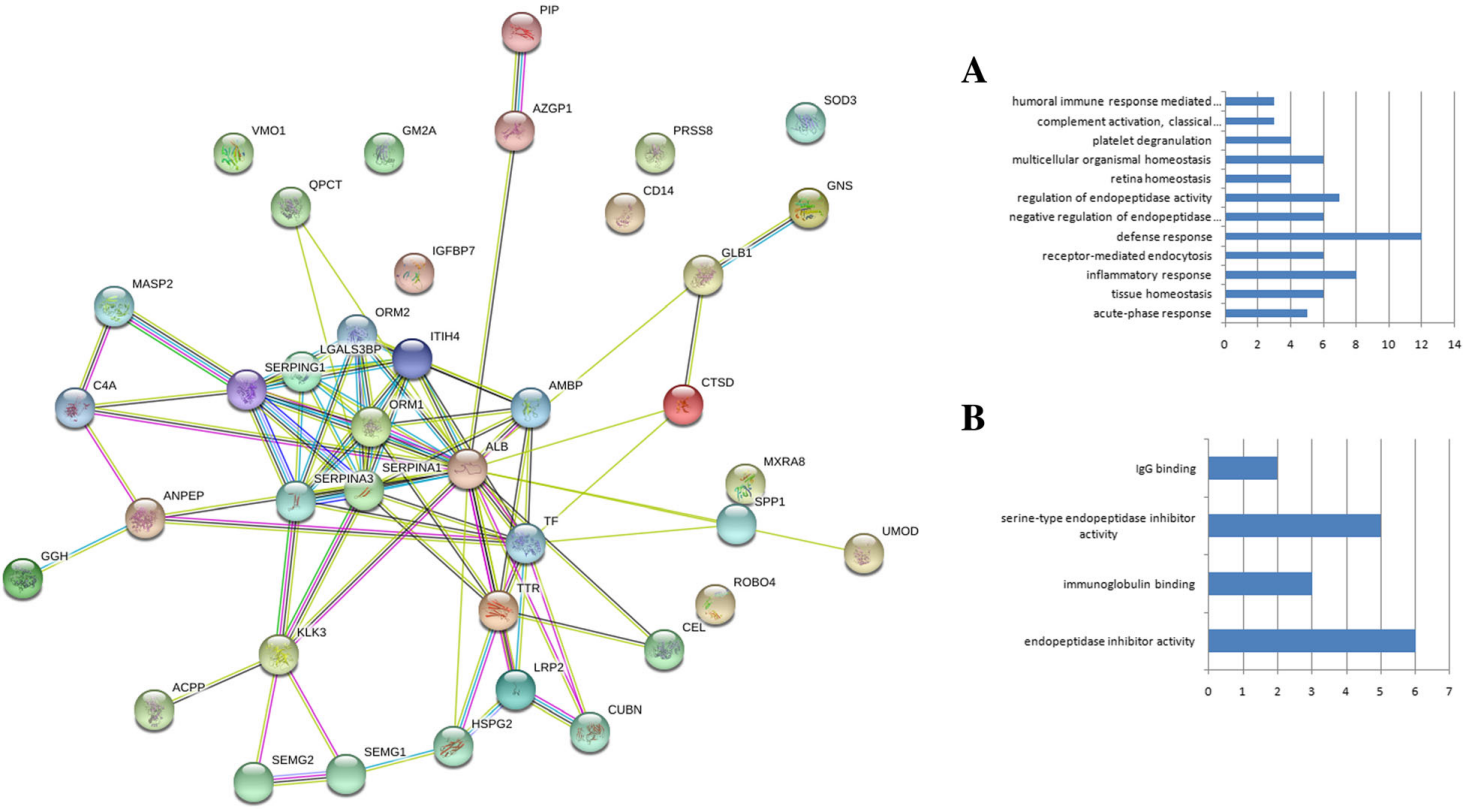
Proteins association network constructed between urine proteins that are significantly changed after long-term spaceflight: **a** biological processes; **b** molecular function

Most of the proteins are participating in defense and inflammatory response, and are involved in homeostasis. Proteins association network shows associations between proteins that are significantly changed after long-term spaceflight (Fig. 2).

## Conclusion

Thus, the above-described urine proteome changes are significantly different in the groups identified by heart rate autonomic regulation peculiarities before space flight. All these proteins regulate the associated biological processes which affect the stiffness of the vascular wall, blood pressure level, the severity of atherosclerotic changes, the rate and degree of age-related involution of elastin and fibulin, age-related increase in collagen stiffness, genetically determined features of elastin fibers. The increased vascular rigidity (including the aorta) and of myocardium may be regarded as a universal response to various extreme factors.

The influence of space flight factors, the use of drug preventive measures and of various physical training modes forces the body to change constantly, providing an adequate level of the main vital systems functioning. The plasticity of the homeostasis systems is determined genetically. The observed sympathetic and parasympathetic activity is the result of a multi-loop and multi-level response of the circulatory regulation system, which changes its parameters continuously to achieve an optimal adaptive response, which reflects the adaptive response of the whole organism. Significant differences in the semi-quantitative analysis of signal proteins between groups with different types of autonomic regulation are explained by a common goal: to ensure optimal adaptation regardless of age and of the genetically determined type of responses to the extreme environmental factors effects.

## Additional files

Additional file 1: Electrocardiogram (ECG) data correlation with urine-proteins of two groups with different type of autonomic regulation. (XLS 185 kb)

Additional file 2: Label-free data for urine-protein list of two groups with different type of autonomic regulation. (XLSX 58 kb)

## Abbreviations

ECG: Electrocardiogram
ECM: Extracellular matrix
EPCR: Endothelial protein receptor C
HR: Heart rate
HRV: Heart rate variability
IPSP: Plasma serine protease inhibitor
ISS: International space station
SF: Space flights
TTR: Transthyretin
